# Retraction: AR-v7 protein expression is regulated by protein kinase and phosphatase

**DOI:** 10.18632/oncotarget.28794

**Published:** 2025-12-31

**Authors:** Yinan Li, Ning Xie, Martin E. Gleave, Paul S. Rennie, Xuesen Dong

**Affiliations:** ^1^Vancouver Prostate Centre, Department of Urologic Sciences, University of British Columbia, Vancouver, Canada

**This article has been retracted:** Oncotarget has concluded its investigation of this article. There are several concerns regarding image duplications and manipulations in the manuscript. Specifically, the Actin band in the 293T cell Western blot panel of Figure 2C is a duplicate of the Actin band shown in the 22Rv1 cells panel of the same figure.

Furthermore, the total Western blot panels for the LN95 and 22Rv1 cell lines have been reproduced in Figure 3C. However, the Western blot for PP-1 in 22Rv1 cells was changed, and there is no explanation provided for this change or the reproduction itself.

In Figure 5A, the Western blot of AR-v7 for the AR-v7 S424A mutant has been cropped and flipped for its use in Figure 5B to represent the AR-v7 in S213A mutant. Additionally, the Actin Western blot in Figure 5E was reused as a p38 Western blot in an unrelated later publication by the same authors and has since been corrected [1].

Regarding the supplementary figures, we observed a duplication between the LNCap AR-FL bands in Supplementary Figure 2A and the PC3 AR-v7 bands in Supplementary Figure 2B. Moreover, the PC3 cells AR-v7 Western blot in Supplementary Figure 5B is a duplication of the Actin Western blot for 293T cells presented in Supplementary Figure 7.

The authors initially cooperated with the investigation and provided several original blots, but these lacked the required datestamps. Critically, there was insufficient original data provided to support all necessary corrections. Given the extent and number of required corrections, which exceeded a reasonable limit, the Editorial decision was made to retract the publication. The authors have been informed of this decision but have indicated they do not agree with the retraction.

Original article: Oncotarget. 2015; 6:33743–33754. 33743-33754. https://doi.org/10.18632/oncotarget.5608
